# Community Health Worker Use of Smart Devices for Health Promotion: Scoping Review

**DOI:** 10.2196/42023

**Published:** 2023-02-22

**Authors:** Merlin Greuel, Frithjof Sy, Till Bärnighausen, Maya Adam, Alain Vandormael, Jennifer Gates, Guy Harling

**Affiliations:** 1 Heidelberg Institute of Global Health Heidelberg University Heidelberg Germany; 2 Africa Health Research Institute KwaZulu-Natal South Africa; 3 Department of Global Health and Population Harvard TH Chan School of Public Health Harvard University Boston, MA United States; 4 Department of Pediatrics School of Medicine Stanford University Stanford, CA United States; 5 Icahn School of Medicine at Mount Sinai New York, NY United States; 6 Institute for Global Health University College London London United Kingdom; 7 Department of Epidemiology and Harvard Center for Population and Development Studies Harvard TH Chan School of Public Health Harvard University Boston, MA United States; 8 Medical Research Council/Wits Rural Public Health and Health Transitions Research Unit (Agincourt) Faculty of Health Sciences University of the Witwatersrand Johannesburg South Africa; 9 School of Nursing and Public Health College of Health Sciences University of KwaZulu-Natal KwaZulu-Natal South Africa

**Keywords:** mobile health, community health workers, smart phones, tablets, health promotion, public health, health worker, smart devices, health behaviour, smart technology, health message, health outcome

## Abstract

**Background:**

Community health workers (CHWs) have become essential to the promotion of healthy behaviors, yet their work is complicated by challenges both within and beyond their control. These challenges include resistance to the change of existing behaviors, disbelief of health messages, limited community health literacy, insufficient CHW communication skills and knowledge, lack of community interest and respect for CHWs, and CHWs’ lack of adequate supplies. The rising penetration of “smart” technology (eg, smartphones and tablets) in low- and middle-income countries facilitates the use of portable electronic devices in the field.

**Objective:**

This scoping review examines to what extent mobile health in the form of smart devices may enhance the delivery of public health messages in CHW-client interactions, thereby addressing the aforementioned challenges and inducing client behavior change.

**Methods:**

We conducted a structured search of the PubMed and LILACS databases using subject heading terms in 4 categories: technology user, technology device, use of technology, and outcome. Eligibility criteria included publication since January 2007, CHWs delivering a health message aided by a smart device, and face-to-face communication between CHWs and clients. Eligible studies were analyzed qualitatively using a modified version of the Partners in Health conceptual framework.

**Results:**

We identified 12 eligible studies, 10 (83%) of which used qualitative or mixed methods approaches. We found that smart devices mitigate challenges encountered by CHWs by improving their knowledge, motivation, and creativity (eg, through self-made videos); their status within the community; and the credibility of their health messages. The technology stimulated interest in both CHWs and clients—and sometimes even in bystanders and neighbors. Media content produced locally or reflecting local customs was strongly embraced. Yet, the effect of smart devices on the quality of CHW-client interactions was inconclusive. Interactions suffered as CHWs were tempted to replace educational conversations with clients by passively watching video content. Furthermore, a series of technical difficulties experienced especially by older and less educated CHWs compromised some of the advantages brought about by mobile devices. Adequate CHW training ameliorated these difficulties. Only 1 study (8%) considered client health behavior change as an end point, thus revealing a major research gap.

**Conclusions:**

Smart mobile devices may augment CHWs’ field performance and enhance face-to-face interactions with clients, yet they also generate new challenges. The available evidence is scarce, mostly qualitative, and focused on a limited range of health outcomes. Future research should include larger-scale interventions across a wide range of health outcomes and feature client health behavior change as an end point.

## Introduction

**Figure 1:**
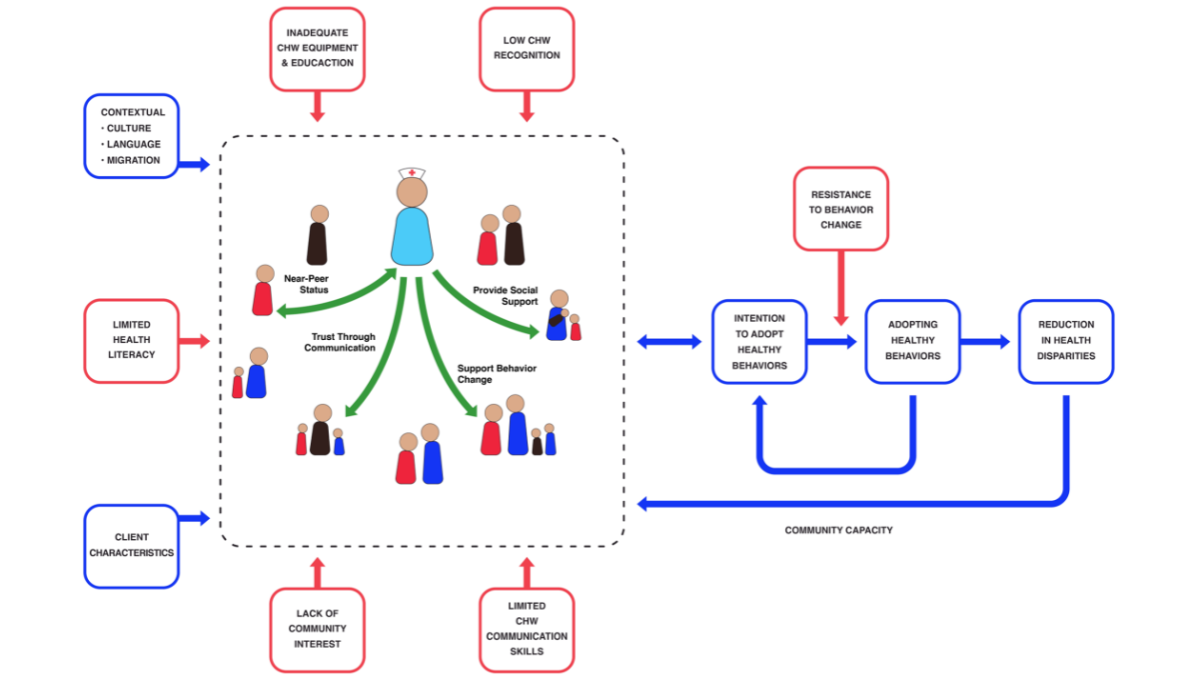
Conceptual framework of facilitators and barriers to community health workers (CHWs) and patients acting as partners in health. Challenges to the CHW-client interaction are shown in red (adapted from Katigbak et al [3] with permission from the American Journal of Public Health).

Community health workers (CHWs) have become central to health promotion activities, with more than 5 million CHWs working worldwide in 2014 [1]. In part, CHWs’ impact is a result of speaking the local language and identifying with the community they serve. Therefore, they have the potential to convey health messages more effectively than other health cadres [2] and may be able to “improve key health-related behaviors” [1]. Katigbak and colleagues [3] have developed the Partners in Health conceptual framework for how CHWs can facilitate the adoption of healthy behaviors. In this framework, client characteristics, the environment, and CHW activities reciprocally influence each other to generate behavior change. Nevertheless, factors both within and beyond CHWs’ control can impede their health promotion activities. Based on the literature cited below, we have identified challenges to CHW health promotion activities and have integrated them into the Partners in Health framework (Figure 1).

One challenge is the way humans manage change, as promoting healthy behavior often entails encouraging changes in existing behavior. Since multiple social, emotional, and cognitive factors interact to mediate [4] and sustain behavior change [5], harmful behaviors are often resistant to change. A second challenge to promoting healthy behaviors is community literacy. In particular, limited health literacy, the ability to comprehend and act on health-related information, is associated with negative health outcomes [6,7] and may complicate health message uptake. In contrast, adequate health literacy can promote healthy behaviors, such as physical activity, by increasing knowledge and self-efficacy related to these behaviors, resulting in positive health outcomes [7]. While low health literacy is certainly a problem in higher-income countries [6], it constitutes a larger problem in low- and middle-income countries (LMIC). For example, basic literacy in many sub-Saharan African countries ranges from 24% to 60% [8].

Other challenges relate to the characteristics of CHWs and their interaction with community members. These include insufficient CHW communication skills [9]. In addition, a lack of community participation and interest, CHWs’ own limitations in understanding complex health information due to low levels of education, a lack of respect for CHW knowledge, and disbelief in health promotion messages may complicate the work of CHWs [10]. The lack of community recognition and the low community status of CHWs may pose additional challenges [11], and this problem may be aggravated if CHWs lack adequate supplies and equipment [12]. Facing these challenges, CHWs have demanded educational communication materials that can be carried to the households they visit [9] and suggested using media to reinforce health messages [10].

Mobile electronic media—in particular “smart” devices such as smartphones and tablets—may constitute powerful tools to deliver public health messages. Smart devices can provide learning via videos or mobile apps, providing information through multiple modes (eg, verbally and visually). Learners presented with visual information in addition to verbal information generate a multimedia effect that deepens learning [13]. Dual coding theory suggests that this deeper learning occurs because learners process visual and verbal information separately and then select pieces of information from each before unifying them into a coherent mental representation of knowledge [14]. This theory has been used to optimize multimedia learning materials for e-learning [15] and medical education [16].

The rapidly rising smartphone ownership in LMIC [17] presents an opportunity for increased access to health-related information and resources extending health system reach [18]. However, the comparatively low penetration of smart devices in low-income settings may limit their usefulness as health promotion vehicles; in several sub-Saharan African countries, adult smartphone ownership is less than 20% [17]. Equipping CHWs with mobile smart devices provides an intermediate solution, allowing electronic multimedia education to be accessed even in low device-penetration communities. For example, tablet-displayed videos have transmitted agricultural knowledge and induced abstractive learning in rural Uganda [19].

Given the potential of smart devices as health promotion vehicles, equipping CHWs with such devices may address several of the aforementioned health promotion challenges. Past reviews of mobile health (mHealth) and CHWs have not focused specifically on the use of mHealth as a health promotion tool. Braun and colleagues’ [20] review of CHW mHealth use concentrated on how mHealth improved intra-CHW communication and learning. Hall and colleagues’ [21] review of mHealth interventions in LMIC included client education and behavior change but focused on the role of mHealth in improving treatment adherence and appointment compliance rather than multimedia applications. Källander and colleagues’ [22] review considered SMS text messaging rather than multimedia applications, while the review by White et al [23] focused on how mHealth improved CHW-patient communication broadly but did not focus specifically on educational uses. Thus, there has not been a systematic review of how smart mobile devices can facilitate the delivery of public health messages by CHWs. 

Accordingly, we conducted a scoping review of how multimedia features of smart mobile devices have been used to enhance knowledge transfer and behavior change by CHWs. We excluded distance-based media approaches such as SMS text messaging, automated voice messages, and phone calls and focused instead on studies involving direct face-to-face communication between CHWs and community members. Our fundamental question is whether smart mobile devices can enhance CHW face-to-face delivery of public health messages and thereby enhance client health behavior change. The aim of this study was to identify the type of evidence available, point out knowledge gaps, and indicate possible directions for future research. Given these objectives, we deemed a scoping review approach as the most suitable [24].

## Methods

Following the PRISMA-ScR (Preferred Reporting Items for Systematic Reviews and Meta-analyses extension for Scoping Reviews) methodology [25], we used a scoping approach to review the literature published between January 1, 2007, and January 5, 2022. The start date was chosen because the first publicly available smartphones using capacitive touch screens were released in 2007 (tablet computers became more common after 2010). The search was performed in English, but no articles were excluded from the full-text assessment if they were published in another language. We consulted the PubMed and LILACS databases, modifying White and colleagues’ [23] strategy to capture the intersection of 4 search categories: technology user, technology device, use of technology, and outcome.

We defined our “technology users” as CHWs using smart devices to deliver public health messages and client recipients. As definitions for CHWs vary [26], we employed the common definition used in a World Health Organization study group review, in that CHWs should be “members of the communities where they work, should be selected by the communities, should be answerable to the communities for their activities, should be supported by the health system but not necessarily a part of its organization, and have shorter training than professional workers” [27]. Clients are defined as community and household members of any age or gender who receive a health message outside the context of health facilities.

For “technology device” and “use of technology,” we focused on digital content requiring portable computer-like “smart” devices that distinguish themselves from regular cell phones by the ability to run apps, show video content, and connect to the internet. We excluded technological features not requiring “smart” devices that can be used by traditional cell phones, (eg, SMS text messages, automated voice messages, and phone calls). We also required direct, person-to-person communication between CHWs and clients (as opposed to CHWs sending messages from a distance) since we were interested in whether technology enhances the effectiveness of person-to-person communication. The person-to-person communication had to be primary health education (ie, the delivery of a preventative health message such as the promotion of healthy behaviors). We excluded secondary prevention messages such as treatment dissemination or medication reminders. Finally, we included any qualitative or quantitative “outcome” that allowed us to assess the effectiveness, advantages, or disadvantages of the use of smart devices for health promotion from the viewpoint of any stakeholder.

For each category, we identified relevant Medical Subject Headings (MeSH) for PubMed and the corresponding multilingual Descriptores en Ciencias de la Salud in LILACS. The complete search process including all MeSH terms used is shown in Multimedia Appendix 1. For each article fulfilling the inclusion criteria, we screened all references for other potentially relevant articles and used Google Scholar to search for relevant publications citing these included studies, as well as additional publications by the same authors.

Details of all articles found through these searches were extracted to a spreadsheet and deduplicated. Two authors (MG and FS) independently screened the article titles and abstracts for relevance with respect to the inclusion criteria defined above. If deemed relevant by at least 1 of the 2 authors, the article was included in the following stage of review. The same authors then independently reviewed the full text of all retained articles from the abstract screen. All full-text articles deemed relevant by at least 1 of the 2 authors were then discussed in-depth to reach the final agreement on inclusion. Disagreements were resolved through mutual consent or consultation with a third author (GH). We grouped all included studies by methodology, extracting methods and findings into tables, and used these to qualitatively describe the literature in the context of our original conceptual framework. The PRISMA-ScR checklist [25] that guided our approach can be found in Multimedia Appendix 2.

## Results

### Identified Articles

**Figure 2:**
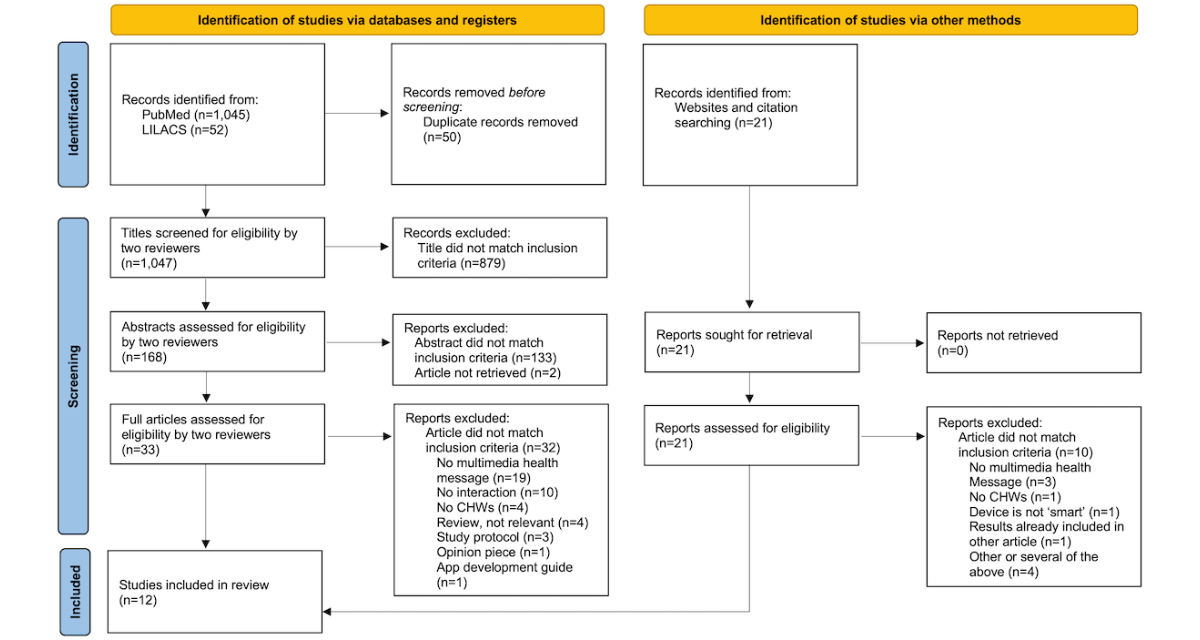
PRISMA (Preferred Reporting Items for Systematic Reviews and Meta-analyses) flow diagram for review articles. Reasons for exclusion do not sum to total because some categories overlap. CHW: community health worker.

The PubMed search yielded 1045 results, and the LILACS search 52, all but 2 (96%) of them duplicates of PubMed articles. Of all articles in either database, 168 articles (16%) were included after the title review, 33 (3%) after the abstract review, and 4 after the full paper review. Of these, 1 was chosen to be included in the results of this publication. From the Google Scholar search and reference screenings, 21 additional abstracts were selected, 11 (52%) of which met our inclusion criteria. All 11 studies were included. Hence, of the 1118 initially identified studies (1068 after deduplication), 12 (1%) were retained for analysis (Figure 2 [28]).

### Overview of Studies

The included studies—all published between 2010 and 2021—were conducted in South Africa [29,30], Nigeria [31], Burkina Faso [32], Lesotho [33-35], and India [36-40] (Table 1). Among them, 9 (75%) studies were rural, 2 (17%) urban, and 1 (8%) both. Moreover, 2 (17%) studies were quantitative, 7 (58%) were qualitative, and 3 (25%) were a combination of both. Five (42%) articles were published in peer-reviewed journals, and 7 (58%) were conference papers. Eight (67%) studies addressed maternal and child health (MCH), 1 (8%) addressed polio immunization, and 3 studies (25%) included multiple health themes. Moreover, 11 (92%) studies included between 7 and 81 CHW participants. Of these, CHWs were the sole or primary participants in 6 (50%) studies; in other cases, they constituted 1 group of participants, alongside mothers, field staff, or mobile shop/laptop owners. The remaining 1 (8%) study examined only clients. Note that sample sizes reported both in the text and Table 1 refer to the participants relevant to our research question and in some cases do not reflect the overall sample size of all participants featured in the study.

**Table 1:** Overview of studies.

Citation	Title	Health issue	Location	Sample	Study type	Publication
Adam et al, 2021 [30]	Evaluation of a community- based mobile video breastfeeding intervention in Khayelitsha, South Africa: The Philani MOVIE^a^ cluster-randomized controlled trial	Maternal and child health (exclusive breastfeeding)	South Africa (urban)	1502 pregnant women	Quantitative (RCT^b^)	Journal
Birukila et al, 2016 [31]	Reducing resistance to polio immunisation with free health camps and Bluetooth messaging: An update from Kaduna, Northern, Nigeria	Resistance to polio immunization	Nigeria (rural and urban)	12,418 households	Quantitative	Journal
Coetzee et al, 2018 [29]	Community health workers’ experiences of using video teaching tools during home visits—A pilot study	Maternal and child health (HIV, alcohol, nutrition, and breastfeeding)	South Africa (urban)	24 CHWs^c^	Qualitative	Journal
Gopalakrishnan et al (2020)	Using mHealth to improve health care delivery in India: A qualitative examination of the perspectives of community health workers and beneficiaries	Maternal and newborn health	India (rural)	32 CHWs, 55 clients	Qualitative (interviews)	Journal
Isler et al, 2019 [32]	Iterative adaptation of a mobile nutrition video-based intervention across countries using human-centered design: Qualitative study	Maternal and child health (nutrition during pregnancy and breastfeeding)	Burkina Faso (rural)	CHWs, mothers (varying N)	Qualitative (focus groups, interviews, observations)	Journal
Kumar et al, 2015 [37]	Projecting health: Community-led video education for maternal health	Maternal and newborn health	India (rural)	CHWs, mothers, field staff (N not specified)	Qualitative (observations, interviews, focus groups)	Conference paper
Molapo et al, 2017 [33]	Video consumption patterns for first-time smartphone users – community health workers in Lesotho	Various	Lesotho (rural)	42 CHWs	Qualitative (observations, interviews, focus groups), quantitative (video views)	Conference paper
Molapo et al, 2016 [34]	Designing with community health workers: enabling productive participation through exploration	Various	Lesotho (rural)	54 CHWs	Qualitative (discussions, focus groups, workshops)	Conference paper
Molapo and Marsden, 2013 [35]	Software support for creating digital health training materials in the field	Various (eg, tuberculosis, sexual health)	Lesotho (rural)	15 CHWs	Qualitative (observations, interviews, focus groups, video logs)	Conference paper
Ramachandran et al, 2010 [38]	Mobile-izing health workers in rural India	Anemia and maternal health	India (rural)	7 CHWs	Qualitative (interviews, observations) and quantitative	Conference paper
Treatman and Lesh, 2012 [39]	Strengthening community health systems with localized multimedia	Maternal health, child nutrition, newborn health	India (rural)	8 CHWs	Qualitative (interviews)	Conference paper
Vashistha and Kumar, 2016 [40]	Mobile video dissemination for community health	Maternal and newborn health (birth preparedness, hand washing, exclusive breastfeeding, thermal care, delayed bathing)	India (rural)	84 mobile phone shop owners, 71 laptop owners, 81 CHWs	Quantitative (number of phone calls) and qualitative (interviews, focus groups, discussions)	Conference paper

^a^MOVIE: Mobile Video Intervention for Exclusive Breastfeeding.

^b^RCT: randomized controlled trial.

^c^CHW: community health worker.

### Quantitative Assessments

Birukila et al [31] assessed the acceptance of videos containing messages to promote polio immunization in rural and urban Nigeria. The videos—described as pictorial, digitalized flipcharts—were shown to parents and caregivers in 21,242 households on CHWs’ smartphones. Almost all (99.9%) of the 11,612 caregivers who watched the videos claimed that these videos met their health information needs, and 85.4% of the 12,418 mobile phone owners agreed to receive the videos via Bluetooth. Over the study period, CHWs shared the videos around 100 times a day.

The only randomized controlled trial (RCT) we encountered was the intervention by Adam et al [30] in South Africa, wherein 1502 pregnant mothers were randomized into 2 groups. The control group received standard of care (SOC) home-based infant feeding counseling by CHWs. Using tablets, the intervention group was shown videos on infant feeding in addition to the SOC. No differences in behavior (infant feeding practices) were observed between the groups at 1 month and 5 months follow-up, but the videos had replaced around 40% of the CHWs’ face-to-face counseling, thereby freeing up time for other health-related tasks. The small increase in maternal knowledge, observed at the 1-month follow-up, was no longer present after 5 months.

### Qualitative and Mixed Assessments

Ramachandran et al [38] evaluated portable multimedia content in rural India. In their study, 7 CHWs used smartphones to show educational videos on maternal health and anemia to pregnant women during weekly household visits. Some of the material was produced by CHWs and featured influential community members. CHWs approached the videos with enthusiasm, yet older CHWs struggled with the technical features of the smartphones. The devices were often used in a noninteractive manner due to a lack of training. However, CHW coaching mitigated these issues. A written test administered before and after the intervention revealed improvements in CHW knowledge of pregnancy danger signs and self-efficacy after the intervention.

Treatman et al [39] developed a smartphone app featuring culturally appropriate color illustrations and audio recordings in the local language containing health messages about topics in MCH. In their study, 8 CHWs in rural India tested the app and were then interviewed. The audio messages were considered more significant than the illustrations; an engaging speaker was deemed especially important. CHWs described the devices as fun to use and impressive to clients, who considered the health messages credible and trustworthy. CHWs preferred the phones over other job aides since they were easier to carry. However, CHWs doubted the effectiveness of the multimedia content if presented without facilitation and thus highlighted the importance of interaction with clients. Moreover, smartphones appeared inept for use in noisy environments or with groups of clients.

In urban South Africa, Coetzee et al [29] provided 24 CHWs with tablets to show videos to pregnant women and mothers during home visits. Pre- and postintervention focus groups were conducted. The tablets increased CHW motivation by amplifying the perceived importance of their work. The videos stimulated clients' interest and attention, improved CHW credibility and time efficiency, and triggered interest even among nontargeted household members. However, some CHWs worried about tablet theft and their credibility and social status being compromised by insufficient technologic capability. Sometimes, tablets were regarded as a means to avoid interaction with clients, especially when CHWs were tired. Moreover, some clients were concerned that the tablets might be recording them, compromising confidentiality.

Molapo et al [33-35] carried out a series of qualitative assessments based on interviews, focus groups, and observations in Lesotho, one of which [33] also contained a quantitative component. In the first intervention [35], a computer application allowed rural trainers of CHWs to create educational videos with local content transferable to the smartphones of 15 CHWs via Bluetooth. Repeated video views helped CHWs deepen their knowledge, and CHWs requested video material deemed especially important. Surprisingly, CHWs not only used the videos for their own education, as was intended, but they also shared them with community members and peers who did not possess smartphones. The health workers experienced a sense of pride, respect from others, and empowerment, and the videos helped them talk about topics that made them feel uncomfortable, such as sexual health.

During the second intervention [34], the existing video content was improved through community feedback. Since CHWs had started showing the videos to community members, their trainers created videos catered to this purpose. Different versions of the application were tested in the field by 54 CHWs, each equipped with a smartphone. CHWs usually showed the videos to groups of clients; they disliked pausing the videos for feedback or questions because the interruptions limited their perceived professionality.

In the last of the 3 interventions, Molapo et al [33] analyzed the results of their 17 months of fieldwork both qualitatively and quantitatively using log data of video views. The 42 CHWs preferred to watch the videos to completion and interact with their clients afterward instead of pausing the videos. Older CHWs handled the smartphones as well as their younger peers after appropriate instructions. In general, CHWs found the smartphones easy to use, though a lack of English literacy sometimes caused problems. Explicit graphic images, such as in videos about sexually transmitted infections, were popular and triggered discussion. The average number of views per video per CHW declined 24% to 87% percent in 16 months, which the authors attribute to a waning novelty effect. However, video views tended to increase shortly after CHW educational workshops, with views increasing for around 3 months. The number of views per video depended on the video’s perceived importance and individual features, with a preference for videos showing influential community members.

In the study by Kumar et al [37] spanning 24 months, Indian CHWs showed educational videos on MCH to community members in 84 rural villages during monthly group gatherings. These events became so popular that CHWs began to organize them independently, thereby exceeding the researchers’ expectations. The videos were played on small, battery-powered projectors; they facilitated CHWs’ explanations, generated discussions, and highly elevated their social status. The latter was especially true for videos starring CHWs, giving them “celebrity status” [37]. Locally filmed videos were the most popular, as community members could relate to the content. Other advantages included the videos' repeatability and credibility boost for health messages.

Vashistha et al [40] attempted to identify the most effective means of distributing offline health videos on personal mobile phones. They equipped 81 CHWs, 84 mobile shop owners, and 71 laptop owners in rural India with videos promoting MCH. The videos featured unique phone numbers, and viewers were urged to call if they liked the video. The number of calls was recorded, and callbacks were conducted to gather viewers’ opinions. By the end of the 14-week study period, mobile shop owners had distributed the video material to 6 times as many clients compared to laptop owners and CHWs. However, the number of calls received from videos distributed by CHWs far exceeded the calls from those disseminated by mobile shop or laptop owners. The authors provided 3 reasons for this finding: the CHWs had stronger ties with their clients, they were considered experts in their domain, and they seemed to have emphasized the importance of making the phone calls. CHWs regarded the technology as an effective way to enable clients to learn, review, and share health-related knowledge.

Gopalakrishnan et al [36] developed a software for smartphones and tested how its use would affect CHW-client interactions. In their study, 32 CHWs showed videos with health messages on MCH to 55 clients during home visits. Postintervention interviews revealed a divergence in the software’s perceived utility between CHWs and beneficiaries; some initial technical difficulties notwithstanding, the former reported increases in CHW authority, the credibility of health messages, client attention, and the involvement of key household decision makers. They also noted a positive impact on behavior change (vaccination rates). However, for most beneficiaries, the software failed to improve interactions with CHWs. On the contrary, CHW-client interactions were harmed by rushed and short visits and the failure of CHWs to mediate interactions appropriately.

Finally, Isler et al [32] adapted a series of videos related to MCH and nutrition from 1 cultural setting (South Africa) to another (Burkina Faso). Animated videos with South African content and design received input from Burkanese CHWs and clients and were then modified to represent the Burkina Faso cultural, linguistic, and physical setting. Clients emphasized that the characters portrayed should reflect community members in appearance, behavior, and financial situation. Moreover, they recommended acknowledging local household structures and hierarchies during video presentations. The ease of tablet usage varied by CHW education and age. Some CHWs expressed technical concerns and preferred reducing the amount of information transmitted in each video viewing session.

## Discussion

### Principal Findings

Studies to date have been conducted in India and across Africa and have largely evaluated the practicability of smartphones or tablets as health promotion vehicles. Despite the diversity of study designs and cultural contexts, the studies have common findings regarding both the ability of mHealth to alleviate some challenges encountered by CHWs and some key drawbacks, such as equivocal effects on CHW-client interactions and frequent technical difficulties in the field. While locally produced media content proved popular, the potential of smart devices as catalysts for health behavior change remains elusive and merits larger-scale, quantitative interventions in the future.

### Overcoming Challenges

mHealth technologies may mitigate some of the challenges experienced by CHWs in the promotion of healthy behaviors. mHealth can increase CHW health knowledge [35,38], thereby addressing CHWs’ own educational deficits. CHWs themselves stressed that the ability to carry mobile videos helped them remember crucial health concepts [35], an assertion verified objectively in a written test [38]. Increased CHW health knowledge should benefit their interactions with clients and increase clients’ respect for the health workers and their work.

As a novel technology, mHealth use by CHWs may stimulate community interest [29-32,35-37,39]. Clients, bystanders, and neighbors were interested mHealth deliveries [29,39], as were CHWs not possessing electronic devices [35]. While this interest may wane over time as recipients become accustomed to the technology, it appears possible to rekindle interest through the introduction of new material [33]. Constant innovations (in the form of new videos, apps, etc) may therefore be necessary to maintain interest. The rising personal ownership of mobile electronic devices in LMIC may limit such novelty effects in the future, making the case for constant technological or creative progress even stronger.

Part of the creativity required for this progress may well be shown by the CHWs themselves, as the introduction of the technology motivated them [29,38] and was overall enjoyable [39]. CHWs became innovative in the development of new media [38] and took ownership of the technology, for example, by independently organizing events going beyond the aims set out by the researchers [37]. At times, CHW motivation also extended to influential community members who became involved in the projects [38]. The increased levels of CHW motivation may be explained to some extent by their elevated levels of self-efficacy [38] and community recognition [29,30,35-37]. The mHealth technology gave CHWs a sense of pride and empowerment [35] and elevated their social status [30,37]. This, in turn, affected the extent to which clients accepted health messages, as they considered them trustworthy and credible [29,30,37,39]. Thus, by boosting CHW motivation, self-efficacy, and community status and by raising the credibility of health messages, mHealth may help CHWs promote healthy behaviors more effectively in their interactions with clients. However, the question remains whether these interactions improved with the use of mobile technology.

### CHW-Client Interactions

The impact of mHealth on CHW-client interactions was ambivalent. On the one hand, CHWs sometimes viewed smart devices as complementary to their interaction with clients [39]. For instance, CHW-moderated group video sessions generated considerable discussion [37]. Devices were particularly beneficial for sensitive topics such as sexual health [35]. On the other hand, CHWs also used portable media to replace client conversations [29,30,38], for example, because they did not know what else to do [38], they were tired [29], or they wanted to save time [30]. In other cases, CHWs preferred to interact with clients only after the videos had played until completion [33,34]. Beneficiaries frequently considered video-assisted health promotion sessions as rushed or too short [36]. In 1 study [36], there was a considerable divergence in how CHWs and clients assessed the use of smartphones; while CHWs embraced them, beneficiaries criticized the quality of interactions. This suggests that mHealth-facilitated health promotion may simplify CHWs’ work by replacing discussions with screen time—at the expense of clients’ experience.

Personal interaction with clients is one of the factors that distinguish CHWs from other health-promoting agents. It is thus questionable whether mHealth can benefit CHWs’ health-promoting efforts should the quality of face-to-face interactions be undermined. However, appropriate CHW training in how to use videos to stimulate discussions may reduce or eliminate the risk of noninteraction, as 1 study showed [38]. Further research should focus on mHealth’s influence on the quality of CHW-client interactions and how a stimulating synergy between technology and interactions can be achieved.

### Technical Difficulties

Even the best electronic device is of no avail if a CHW lacks the knowledge on how to use it. Some CHWs initially struggled when using portable media [32,36,38]. Younger CHWs generally adapted more quickly than their older colleagues [32,38], and higher education levels facilitated the adoption of the technology [32,38]. Due to the technological challenges, CHWs reported feeling anxious [29], nervous [32], and even worried about their community status being compromised [29,34]. However, the technical difficulties were reduced after CHWs received appropriate training [33,38], and even older CHWs learned to use the devices as effectively as their younger peers [33]. Having overcome the initial problems, CHWs enjoyed using the devices [33,39], considered touchscreens user friendly [33], and preferred portable devices over other, bulkier job aides [39]. Some CHWs reported that their skillful usage of the technology enhanced their perceived authority in the community [30]. Hence, proper CHW instruction is the key to converting smart devices from stressors and sources of discomfort into pleasant companions at work.

### Go Local

Both CHWs and clients highlighted the importance of the media featuring local content [32,35,37-39]. When given the opportunity to create their own educational videos, CHWs chose to include testimonials from influential community members [38]. Videos featuring sequences of CHWs raised their social status in the community and enabled clients to identify with the content [37]. Clients preferred locally filmed videos over those shot in different locations [37]. The inclusion of locally appropriate color illustrations and the local language was also appreciated by clients [32,39], who emphasized that animated video content should resemble the local population in appearance and behaviors [32]. Hence, mHealth promotional strategies should adopt local features to maximize client identification with the material. Locally produced content thus has the potential to affect health behaviors more powerfully than material conceptualized elsewhere.

### Client Learning and Behaviors

Perhaps the most important question in the context of mHealth and health promotion is whether mHealth helps CHWs improve clients’ health behaviors. The reviewed literature contained almost no evidence relating to changes in clients’ behaviors or process measures such as behavioral intent or health knowledge. Both clients [31] and CHWs [29,40] regarded the portable devices as enriching educational tools for the recipients of the interventions. Some CHWs provided anecdotal evidence that the use of the devices could have contributed to the adoption of health behaviors such as vaccinations [36]. However, only 1 (8%) of the 12 studies featured behavior change as an end point [30]. In this long-term RCT, the researchers observed no effect of the mHealth video intervention on health behaviors and only a small positive effect on health knowledge [30]. Thus, the potential of mHealth to bring about behavior change when employed by CHWs remains unclear.

### Looking Ahead

**Figure 3:**
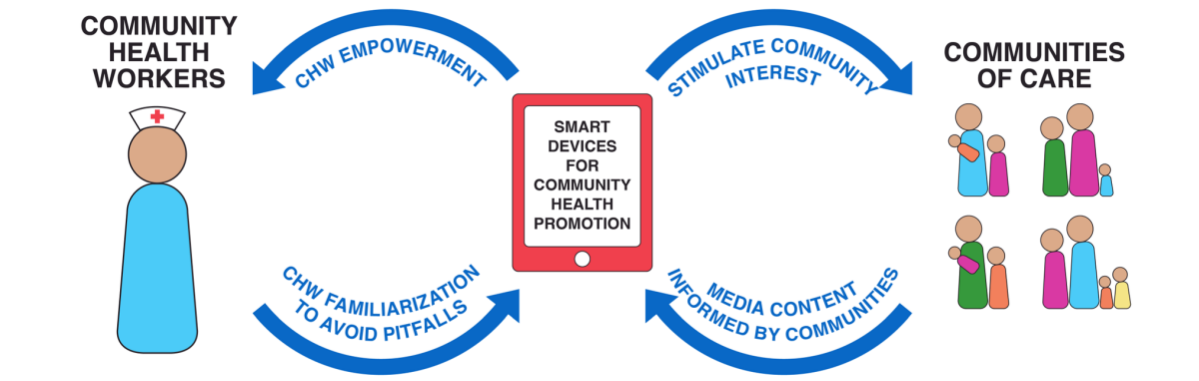
Smart devices as catalysts for community health promotion.

The studies included in this review contain valuable information on the impact of mobile electronic devices on the delivery of health messages by CHWs. mHealth can empower CHWs and potentially help alleviate many challenges faced in the field. By stimulating community interest, health messages may be conveyed more effectively. Media content informed by the targeted communities themselves has been shown to be especially persuasive. However, the use of mobile media will always require careful training to maximize benefits and minimize potential pitfalls. CHWs should be familiarized with the technology and instructed on employing it as a complement to their interactions with clients, not as a replacement thereof. If used appropriately, smart devices may catalyze health promotion, benefitting CHWs and clients alike (Figure 3).

Nevertheless, our primary research question—whether mHealth improves CHW-led face-to-face delivery of public health messages and behavior change interventions—remains unanswered. Notably, all but 2 (83%) of the studies in this review are qualitative. While these are invaluable for planning, improvement, and evaluation, more large-scale quantitative studies, such as the included RCT [30], are needed with behavior change end points. In addition, 8 (67%) of the 12 studies in this review focus on MCH. This reflects the importance of MCH in low-income settings, but a wider scope of mHealth assessments would be desirable.

### Limitations

Our review has some limitations. First, we only used 2 databases which, while wide-ranging in scope, did not capture all the relevant published studies we finally used. Therefore, we may have missed other published studies. Second, capturing all relevant research in this fast-moving field is difficult, as highlighted by most of the included work being only available as conference papers. These findings suggest the importance of future updates to this review.

### Conclusions

Novel technological improvements may increase the effectiveness of CHW-led promotion of healthy behaviors. In this review, we show that smart mobile devices have the potential to enhance face-to-face interactions between CHWs and their clients, as these job aides address many of the challenges that CHWs commonly encounter in the field. However, we also find that the available evidence on our research question is scarce, largely qualitative, and focused on a limited scope of health outcomes. In particular, it is unclear whether mHealth helps CHWs change clients’ health behaviors. Moreover, the impact of employing mHealth in the field is not all positive, as smart devices may burden CHWs with technological difficulties and lead them to act more passively in their interactions with clients. Further research is required to develop interventions to address this issue, along with large-scale quantitative interventions across a wider range of health outcomes to determine the full potential for interactive mHealth interventions to support CHW behavior change work in low-resource settings.

## Appendix

Multimedia Appendix 1 Database search entries.

Multimedia Appendix 2 PRISMA-ScR (Preferred Reporting Items for Systematic Reviews and Meta-analyses extension for Scoping Reviews) checklist.

## Abbreviations

CHW: community health worker
LMIC: low- and middle-income countries
MCH: maternal and child health
MeSH: Medical Subject Headings
mHealth: mobile health
PRISMA-ScR: Preferred Reporting Items for Systematic Reviews and Meta-analyses extension for Scoping Reviews
RCT: randomized controlled trial
SOC: standard of care
