# Supplementary material for: Community Health Worker Use of Smart Devices for Health Promotion: Scoping Review
Source: JMIR Mhealth Uhealth. 2023 Feb 22;11:e42023. doi: 10.2196/42023 (PMC9996418; doi:10.2196/42023)
Supplement: Multimedia Appendix 1 [file mhealth_v11i1e42023_app1.docx]

**Appendix 1: Database search entries.**

**PubMed Search Entry**

(community health workers[Mesh] OR caregivers[Mesh] OR health personnel[Mesh] OR home care services[Mesh] OR maternal health services[Mesh] OR mentors[Mesh] OR patient care team[Mesh] OR peer group[Mesh] OR rural health services [Mesh])

AND

(cellular phone[Mesh] OR computers, handheld[Mesh] OR mobile applications[Mesh] OR user-computer interface [Mesh])

AND

(delivery of health care[Mesh] OR interviews as topic[Mesh] OR public health/education[Mesh] OR remote consultation[Mesh] OR health education[Mesh] OR health promotion[Mesh])

AND

(communication[Mesh] OR health behavior[Mesh] OR health communication[Mesh] OR health knowledge[Mesh] OR patient acceptance of health care[Mesh] OR patient compliance[Mesh] OR quality of health care[Mesh] OR treatment outcome [Mesh] OR life style[Mesh] OR health literacy[Mesh] OR information literacy[Mesh])

**LILACS Search Entry**

(MH:"community health workers" OR MH:"caregivers" OR MH:"health personnel" OR MH"home care services" OR MH:"maternal health services" OR MH:"mentors" OR MH:"patient care team" OR MH:"peer group" OR MH:"rural health services") AND (MH:"cellular phone" OR MH:"computers, handheld" OR MH:"mobile applications" OR MH:"user-computer interface") AND (MH:"delivery of health care" OR MH:"interviews as topic" OR MH:"public health/education" OR MH:"remote consultation" OR MH:"health education" OR MH:"health promotion") AND (MH:"communication" OR MH:"health behavior" OR MH:"health communication" OR MH:"health knowledge" OR MH:"patient acceptance of health care" OR MH:"patient compliance" OR MH:"quality of health care" OR MH:"treatment outcome" OR MH:"life style" OR MH:"health literacy" OR MH:"information literacy")
